# Motivational Strategies, Language Learning Strategies, and Literal and Inferential Comprehension in Second Language Chinese Reading: A Structural Equation Modeling Study

**DOI:** 10.3389/fpsyg.2021.707538

**Published:** 2021-08-12

**Authors:** Lin Lin, Wai-Ip Lam, Shek Kam Tse

**Affiliations:** ^1^School of International Cultural Exchange, Shanghai University of Finance and Economics, Shanghai, China; ^2^Faculty of Education, University of Hong Kong, Hong Kong, China

**Keywords:** motivational strategies, language learning strategies, literal comprehension, inferential comprehension, L2 chinese reading

## Abstract

Motivational strategies have been recognized as a crucial but insufficiently explored component in second language (L2) learning. This study intends to explore the relationships between motivational strategies, language learning strategies, and literal and inferential comprehension in L2 Chinese reading. Data were collected from 547 international students of universities in mainland China through a strategy use questionnaire and a Chinese reading test. The analysis of the structural equation model indicated that motivational strategies indirectly affected literal comprehension through the mediation of learning strategies. Moreover, motivational strategies were found to directly affect inferential comprehension. The results emphasize the need for a more sophisticated analysis of the motivational strategies and language learning strategies in L2 Chinese reading.

## Introduction

Over the last two decades, Chinese has been recognized as an important foreign language (FL) or second language (L2) taught and learnt within and outside China (Gong et al., 2018, 2020c,d). At the end of 2018, more than 492 thousand people from 196 countries and regions were reported learning L2 Chinese in mainland China (Ministry of Education of the People’s Republic of China, 2019). The growing interest of learning Chinese around the world has called for research into Chinese language teaching and learning (Gong et al., 2020a).

One of the issues concerns L2 learners’ strategy use in Chinese language learning. Researchers in L2 education have recognized the vital role of motivational strategies in learning (Oxford, 1990, 2011; Dörnyei, 2005). L2 learners use motivational strategies to initiate their willingness to start learning and sustain their efforts and perseverance in tedious foreign language learning (Cheng and Dörnyei, 2007). In recent decades, there is a growing interest in developing techniques to increase motivation and explaining the relationships between motivational strategies with language learning strategies (e.g., cognition and metacognition) and learning outcomes (Dörnyei and Csizér, 1998; Cheng and Dörnyei, 2007; Teng and Zhang, 2018). However, there lacks empirical clarity concerning how motivational strategies, while interacting with language learning strategies, influence learners’ performance.

Previous research has already found how L2 learners use learning strategies to improve their reading performance (Phakiti, 2003, 2008; Zhang and Zhang, 2013; Zhang et al., 2014). However, most research studies have investigated reading comprehension as a global construct regarding its relationship to language learning strategies (Phakiti, 2003, 2008; Zhang et al., 2014), neglecting the multilevel complexity of comprehension (Kintsch, 1998) when L2 learners interact with texts. Each level of comprehension requires different cognitive demands (Pearson and Johnson, 1978; Kintsch and Rawson, 2005), implying that L2 learners may use various strategies for a specific level of comprehension. Literal and inferential comprehension are two levels of reading comprehension widely used to design comprehension questions in reading tests and recommended in teaching practices and instructional books (Eason et al., 2012; Basaraba et al., 2013). Understanding their relationships with strategy use, including motivational, cognitive, and metacognitive strategies in reading, would help language teachers and learners identify specific strategy use patterns for achieving a certain level of reading comprehension and later adjust their teaching and learning. Given the limited empirical studies on this topic, there is a need to investigate the relationships between L2 learners’ strategy use and the reading performance in literal and inferential comprehension.

Most research studies investigating the effects of strategy use on reading comprehension have been conducted in L2 English contexts (Purpura, 1997, 1999; Phakiti, 2003, 2008; Zhang and Zhang, 2013). To date, few studies have examined the interactions between learners’ strategy use and reading comprehension performance in L2 Chinese. Compared with English reading, Chinese reading involves different cognitive processes and linguistic characteristics (Koda, 2005; Zhou et al., 2018). L2 Chinese learners may employ distinctive strategies to comprehend texts. Research findings on the strategy use in L2 Chinese reading would contribute to the theoretical development on L2 acquisition by supporting, challenging or proposing modifications to the existing knowledge of L2 theories (Han, 2017).

Given these research gaps mentioned above, this study aimed to explore the influences of motivational and language learning strategies on literal and inferential comprehension in L2 Chinese reading. Its purpose was to determine whether certain types of strategies might affect reading performance at a specific comprehension level. The study also examined how motivational studies interacted with language learning strategies in L2 Chinese reading. For the purpose of this study, L2 Chinese specifically refers to learning L2 Chinese in Chinese-speaking environments. Learning Chinese as an additional language or a FL is not the focus of the present study.

## Literature Review

Literature review on strategy use in L2 Chinese reading includes four major parts: motivational strategies in L2 learning, language learning strategies in L2 reading, literal and inferential comprehension in L2 reading, and strategy use and L2 Chinese reading comprehension. The first part addresses the role of motivational strategies in L2 learning and examines the studies that have investigated the relationships between L2 learners’ strategy use and academic performance. Then, it moves on to a review of the development of language learning strategies in L2 reading. After that, the roles of different levels of comprehension in L2 reading ability is discussed. The last part reviews strategy use research in L2 Chinese reading context, which is the main focus of the literature review.

### Motivational Strategies in L2 Learning

Motivation strategies are activities that individuals intentionally perform to initiate, maintain, or increase their willingness to start or complete a specific task or goal (Wolters, 2003). According to Dörnyei (2005), the purpose of motivational strategies in L2 learning is “to generate and enhance student motivation, as well as maintain ongoing motivated behavior and protect it from distracting and/or competing action tendencies” (p. 117). L2 learners use motivational strategies purposefully to influence their choices, efforts, or persistence for academic works and eventually impact their learning outcomes (Wolters, 2003; Pintrich, 2004). Although motivational strategies have been recognized as an essential part of several L2 strategy taxonomies (Oxford, 1990, 2011; Dörnyei, 2005), empirical research on their relations to other types of strategies and L2 learners’ academic performance is still inadequate.

A few studies have tried to explore the effects of motivational strategies on the academic outcomes in self-regulated learning, which is closely related to theories and empirical studies of language learner strategies in L2 learning (Pintrich and De Groot, 1990; Wolters, 1998, 1999; Schwinger and Stiensmeier-Pelster, 2012). Self-regulated learning is an active and constructive process whereby learners set learning goals and monitor, regulate and control themselves cognitively, behaviorally and emotionally to achieve their goals (Pintrich, 2000; Zimmerman, 2002). Self-regulated learners can apply a number of strategies and adapt their behaviors when they encounter problems in language learning (Zimmerman, 2008). Some researchers argued that motivational strategies directly and positively influenced learners’ academic outcomes (Wolters, 1999; Cheng and Dörnyei, 2007). However, other researchers claimed that motivational strategies alone were not enough to influence learners’ learning outcomes and needed to work in combination with other strategies to achieve a significant effect on the academic performance (Pintrich and De Groot, 1990; Pekrun et al., 2007; Mega et al., 2014).

Focusing only on investigating the direct effects of motivational strategies is likely to undermine the importance of motivational regulation on academic achievement as motivational strategies aim to optimize learners’ learning efforts, persistence, or choices of activities (Wolters, 2003; Schwinger and Stiensmeier-Pelster, 2012). Pintrich and De Groot (1990) found that learners’ motivational strategies did not directly affect classroom academic performance but were strongly correlated with cognitive strategies. Schwinger and Stiensmeier-Pelster (2012) reported an indirect effect of motivational strategies on students’ academic performance through the mediation of learning effort.

Schwinger and Stiensmeier-Pelster (2012) also pointed out the lack of investigating the effect of motivational strategies in specific academic disciplines or contexts. Students tend to feel more or less motivated in different academic discipline, which may later influence whether a specific motivational strategy effectively sustains or enhances their learning efforts. For example, students’ cognition of mathematics is different from that of German and English. They believed that mathematics requires more effort and more intelligence than the other two subjects (Haag and Götz, 2012). Students’ perceptions of different subjects or learning domains affect the effects of motivational strategies. A majority of the research on motivational strategies in academic disciplines has been conducted within mathematics, history and English contexts (Rotgans and Schmidt, 2009; Greene et al., 2015; Sinatra and Taasoobshirazi, 2018). Few studies have explored the functions of motivational strategies in L2 Chinese reading. There is a need for more research on how motivational strategies are enacted across a variety of disciplinary contexts.

### Language Learning Strategies in L2 Reading

Learning strategies are both behavioral and mental activities adopted by learners to enhance their language ability in L2 learning (Oxford, 1990). Cognitive and metacognitive strategies are two core components of strategies in L2 strategy taxonomies (Oxford, 1990, 2011; O’Malley and Chamot, 1990; Sheorey and Mokhtari, 2001). Cognitive strategies refer to the behaviors a learner uses to solve specific tasks in the learning process. In the process of L2 reading, researchers have generated three categories of cognitive strategies: comprehending, memory, and retrieval (Purpura, 1999; Phakiti, 2008; Zhang and Zhang, 2013). Comprehending includes the use of skills to understand incoming information and identify valuable items for further processing; memory involves storing meaningful information in long-term memory; retrieval concerns recalling specific information from long-term memory.

Metacognitive strategies are mental activities that a learner intentionally employs to control and regulate their learning process (Paris and Winograd, 1990; Cohen and Upton, 2006). Metacognitive strategies in reading include planning, monitoring, and evaluating (Jacobs and Paris, 1987; O’Malley and Chamot, 1990). Planning strategies refer to previewing tasks and choosing specific activities for pre-set goals; monitoring is concerned with examining ongoing thoughts and actions in the reading process; evaluating involves assessing one’s past, current, and future cognitive actions for reading tasks (Phakiti, 2008; Zhang and Zhang, 2013).

A group of empirical studies have explored how L2 learners’ strategy use is associated with their L2 English reading performance (Phakiti, 2003, 2008; Zhang and Zhang, 2013; Zhang et al., 2014). Phakiti (2003) investigated the relationships between L2 learners’ strategy use and their English reading test performance and found weak and positive relationships of cognitive and metacognitive strategies to reading performance. Later, Phakiti (2008) conducted another strategy use study and discovered that metacognitive strategy use had an indirect influence on reading test performance through the mediation of cognitive strategy use. Cognitive strategy use itself had a direct effect on lexico-grammatical reading ability, which primarily concerns learners’ competence in literal comprehension. Metacognitive strategy use strongly affected on cognitive strategy use. Zhang et al. (2014) discovered that cognitive and metacognitive strategies operated jointly to impact L2 learners’ lexico-grammatical reading ability assessed in College English Test Band 4 (CET-4) reading subtest. They argued that cognitive and metacognitive strategies might work collectively under a unitary construct to improve L2 learners’ reading performance, regardless of their complex characteristics. Although there is an increasing agreement that using these learning strategies improves L2 learners’ English reading performance (Phakiti, 2003, 2008; Zhang and Zhang, 2013; Zhang et al., 2014), there is no consensus on the relationship between cognitive and metacognitive strategy use.

Cognitive and metacognitive strategies are closely related to motivational strategies in the learning process, especially in self-regulated learning. Wolters (1999) discovered that motivational regulation strategies explained 22 and 32% of the variance in learners’ use of metacognitive and cognitive strategies. His later study (2003) revealed that motivational strategies were positively associated with higher cognitive strategies. Similarly, Pekrun (2006) reported that motivational strategies improved cognitive and metacognitive strategy use in academic contexts. These findings may indicate that motivational strategies may serve as an antecedent of, or operate concurrently with, cognitive and metacognitive strategies to help L2 learners improve their academic performance, as argued by some researchers (Wolters, 2003; Pintrich, 2004). Although previous research has indicated that motivational, cognitive and metacognitive strategies promote L2 learners’ reading performance, it is unclear how motivational strategies interact with the other two types of learning strategies to affect reading achievement. Therefore, more empirical research is required.

### Literal and Inferential Comprehension in L2 Reading

Reading comprehension is “the ability to receive and interpret information encoded in language form via the medium of print” (Urquhart and Weir, 1998, p. 22). Reading comprehension involves a complex interaction between bottom-up word-level processing and top-down meaning processing (Rumelhart, 1977; Rayner and Pollatsek, 1989). Levels of comprehension appear in many instructional textbooks recommended for classroom teaching and reading tests in the form of questions to assess learners’ comprehension in first language (L1) and L2 reading research (Eason et al., 2012; Basaraba et al., 2013). Literal comprehension and inferential comprehension are two levels of comprehension that language learners encounter most frequently when they engage in reading.

Pearson and Johnson (1978) described literal and inferential comprehension by introducing three types of reading comprehension questions: textually explicit, textually implicit, and scriptally implicit questions. Textually explicit questions, related to literal comprehension, are used to examine a reader’s understanding when answers are directly located in the text. Textually implicit questions assess readers’ inferential comprehension when making logical inferences about information not explicitly stated in the texts. Scriptally implicit questions assess readers’ inferential comprehension in integrating their background knowledge and experiences with the information described in the text.

Kim (2009) based on previous research (Halliday and Hasan, 1989; Kintsch, 1998), proposed a framework of L2 comprehension levels composed of three categories: literal comprehension, inferential comprehension with endophoric reference, and inferential comprehension with exophoric reference. Inferential comprehension with endophoric reference concerns understanding implicit information from the text, whereas inferential comprehension with exophoric reference refers to comprehending implicit information, combined with extra knowledge outside the text. Both are consistent with Pearson and Johnson’s (1978) classification of reading comprehension questions. In this study, literal comprehension refers to understanding explicitly stated information in the text; inferential comprehension refers to deriving implicit information from the text and integrating information from various parts of the text or prior knowledge and personal experiences.

Previous research has either measured literal reading comprehension only or used a combined measure of literal and inferential reading comprehension, yet has not compared the relationships between strategy use and literal reading comprehension versus inferential reading comprehension (Phakiti, 2003, 2008; Zhang and Zhang, 2013; Zhang et al., 2014). Different levels of comprehension require distinctive cognitive processes and varying degrees of interaction with the texts (Rupp et al., 2006; Alptekin and Erçetin, 2010). Literal comprehension primarily involves linguistic processes, including word recognition, syntactic parsing, and semantic-proposition formation (Grabe, 2009), whereas inferential comprehension involves higher-order processing assesses readers’ competence in interpreting the author’s intended meaning and understanding the underlying message in a group of surface sentences (Dole et al., 1991; Vacca et al., 2009). Through the executive control process in their working memory, readers choose to process certain information strategically and use multiple strategies to achieve reading comprehension in accordance with task difficulties (Grabe, 2014).

Based on the different cognitive-processing demands for answering literal and inferential questions in the reading tasks, previous studies investigating reading comprehension by combining both levels into one construct may have overlooked how strategy use interacts with a specific level of comprehension. Such information may help researchers identify the effectiveness of different strategies used to understand different levels of comprehension. Investigating comprehension performance levels separately will paint a more comprehensive picture of the interactions between L2 learners’ strategy use and their reading performance. Since most previous studies have examined the effects of strategy use on overall reading ability, more empirical studies are needed to investigate how these strategies affect L2 learners’ performance at literal and inferential reading comprehension, respectively.

### Strategy Use and L2 Chinese Reading Comprehension

As a morphosyllabic language, Chinese has a distinct reading process, which is different from English, an alphabetic language. Chinese contrasts clearly to English in the mapping relationships among orthographic representations, morphology and syntax (Peng et al., 2020). Especially in the lower-level reading processing, L2 learners depend heavily on their linguistic knowledge (Shen and Jiang, 2013). L2 learners may use distinctive strategies in reading Chinese texts due to these linguistic and cognitive-processing differences. For example, the written form of Chinese, a character, is independent of its pronunciation, whereas the sound cue usually is identifiable in English (Kong, 2006). Instead of using the strategy of phoneme-grapheme correspondences in English, L2 learners may use semantic and phonetic radical information to retrieve the meanings and sounds of Chinese characters (Zhang et al., 2016). Chinese’s morphological structure is predominantly about compounding in words, rather than inflections and derivations commonly used in English (Koda, 2005; Zhou et al., 2018). L2 learners need to get such information through their understanding of the Chinese text they are reading. Moreover, a Chinese word may contain one or more characters. In written texts, there are no space boundaries between words. L2 learners are required to recognize context-appropriate words with their mental lexicons and grammar knowledge to segment words (Shen and Jiang, 2013; Huang, 2018). However, L2 learners may employ some similar strategies relevant to general reading processes across languages when their proficiency in L2 reading has achieved a certain level (Feng and Mokhtari, 1998; Chuang, 2007). Chuang (2007) compared the strategies used by 345 eighth-grade students when they read English and Chinese texts. The quantitative analysis indicated that high- and average-achievers did not show significant differences in the use of metacognitive, problem-solving and support strategies between English reading and Chinese reading. A similar result was found in Feng and Mokhtari’s (1998) study, in which the majority of strategies identified were used by 20 advanced learners in both English and Chinese reading.

In the past decade, Chinese language teaching and learning as a FL or L2 has attracted more and more attention in and outside China (Gong et al., 2020b, 2021; Ke, 2020). However, there still exists a disparity between the development of L2/FL Chinese and L2 English strategy use research (Jiang and Cohen, 2012; Ma et al., 2017). Most research in mainland Chinese journals still describes L2 learners’ strategy use in Chinese reading through classroom observation and explains well-recognized strategy taxonomies (Jiang and Cohen, 2012). Moreover, most empirical studies have examined strategy use in lower-level reading processes, such as character recognition and word segmentation (Shen, 2004, 2005; Ke, 2020), rather than higher-level processes involving inferential comprehension.

A few studies have examined the interaction between strategy use and L2 Chinese reading performance (Li, 2002; Qian, 2006; Ke and Chan, 2017). Li (2002) recruited 60 intermediate-level L2 learners to complete a questionnaire developed from Oxford’s (1990) theoretical framework of language learning strategies and found no difference in cognitive strategy use between successful and less successful learners in L2 Chinese reading. However, successful L2 learners were identified to use metacognitive strategies more effectively in reading Chinese than their less successful ones. Qian (2006) also used a questionnaire to examine 92 intermediate- and advanced-level Korean learners’ strategy use in reading Chinese texts. She found that the most frequently used reading strategies were predicting and using context. Qian (2006) argued that since Chinese is a context-bound language, these two strategies are effective in Chinese reading. Ke and Chan (2017) examined L2 Chinese learners’ strategy use of different L1 backgrounds across three proficiency levels. They found that L2 learners’ proficiency affected their application of reading strategies. The strategy types improved along with L2 learners’ proficiency levels. However, the number of strategies employed in reading did not differentiate across the three proficiency levels.

Most previous studies on L2 Chinese learners’ strategy use in reading have adopted descriptive or simple inferential analyses, such as binary correlation (Jiang and Cohen, 2012; Ma et al., 2017). These studies fail to examine the causal relationships between L2 learners’ strategy use and their Chinese reading performance. Moreover, the sample size of these studies is relatively small, so their findings are not generalizable to the larger L2 Chinese population. Moreover, most existing empirical evidence of strategy use in reading has been provided by L2 English research. Previous empirical studies have found that using language learning strategies improves L2 learners’ English reading performance, especially their lexico-grammatical reading competence (Phakiti, 2003, 2008; Zhang and Zhang, 2013; Zhang et al., 2014). Since Chinese and English are two different languages with different cognitive processes and linguistic characteristics (Koda, 2005; Shen and Jiang, 2013; Zhou et al., 2018), it is unclear whether L2 Chinese learners use similar strategies as their L2 English counterparts. It is hypothesized that L2 Chinese learners may use language-specific strategies, such as decoding characters and segmenting words, in lower-level reading processes due to the linguistic differences between English and Chinese. However, it is possible that L2 Chinese learners may apply similar strategies as their L2 English counterparts once they reach a certain level of L2 reading proficiency due to the decreasing influence of language-specific factors in reading comprehension (Grabe, 2014).

Although there is an extensive body of literature in these strands contributing to our understanding of strategy use and its relationship to L2 reading comprehension, more research is required with several questions yet to be answered. First, the interrelationships among strategies themselves and their relationships to L2 reading performance are inconclusive. Some studies have found that both cognitive and metacognitive strategies had significant effects on L2 reading test performance (Phakiti, 2003; Zhang et al., 2014); others have revealed that only one type, either cognitive or metacognitive, of strategy use directly influenced L2 reading test performance (Phakiti, 2008; Zhang and Zhang, 2013). In terms of the relationships between cognitive and metacognitive strategies, some researchers have indicated that metacognitive strategy use had an executive function on cognitive strategy use (Phakiti, 2008; Zhang and Zhang, 2013), while others have discovered that cognitive and metacognitive strategy use functioned concurrently in test contexts (Phakiti, 2003; Zhang et al., 2014). Second, the interactions between motivational strategies and language learning strategies and the role of motivational strategies in L2 reading are understood poorly. Third, there is a lack of empirical studies investigating the relationships between L2 learners’ strategy use and their reading performance at different levels of comprehension. Last, few studies have investigated the role of strategy use in L2 Chinese reading.

Considering these gaps, this study addresses two research questions below:

(1) What are the relationships among motivational strategies, learning strategies, and literal comprehension for L2 Chinese learners?
(2) What are the relationships among motivational strategies, learning strategies, and inferential comprehension for L2 Chinese learners?

## Materials and Methods

### Participants

The participants in this study were 547 international students who learned Chinese in universities in mainland China. All the participants were recruited from upper-intermediate level Chinese classes or above in these universities. On average, they had been learning Chinese in mainland China for 2.90 years (*SD* = 1.12) with 1,920 instructional hours at the time of the study. According to the test syllabus of Hanyu Shuiping Kaoshi (HSK), intermediate-level participants who have learned Chinese for approximately two academic years are eligible to take HSK Level 5, in which students are evaluated to use higher-order reading comprehension processes (Chinese Language Council International and Confucius Institute Headquarters, 2009). HSK Level 5 corresponds to Level C1 of the Common European Framework of Reference for Languages (CEFR) (Chinese Language Council International and Confucius Institute Headquarters, 2009). Of all the participants, 353 were female, and 194 were male. The participants’ age ranged from 16 to 38, with a mean of 22.58 and a standard deviation of 5.17. In terms of nationalities, the five largest groups of participants were Korean (*n* = 103), Thai (*n* = 97), Indonesian (*n* = 62), Japanese (*n* = 45), and Russian (*n* = 37). A breakdown of the participants by country is shown in Supplementary Appendix A.

### Instruments

Instruments for this study consisted of a self-reported strategy use questionnaire and a Chinese reading comprehension test. The questionnaire surveyed the motivational strategies and language learning strategies used by the participants. The Chinese reading comprehension test was used to measure their literal and inferential comprehension in L2 reading.

#### Strategy Use Questionnaire

The motivational strategies were measured based on affective strategies of initiating and maintaining motivation conceptualized by Oxford (1990, 2011). Other sources were also consulted in developing the measures (Pintrich and De Groot, 1990; Dörnyei and Csizér, 1998; Dörnyei, 2001, 2005; Cheng and Dörnyei, 2007). The motivational strategies evaluate how frequently students use strategies to initiate and maintain students’ motivation in L2 Chinese reading, such as setting the goals of their learning efforts by getting high grades or improving reading skills and knowledge, avoiding negative assessment of their reading performance, and using thoughts or subvocal statements to enhance their efficacy for an ongoing reading task.

The language learning strategies were categorized into cognitive and metacognitive strategies. Both the cognitive and metacognitive strategies were adapted from Oxford’s (1990) Strategy Inventory for Language Learning (SILL) and Sheorey and Mokhtari’s (2001) Survey of Reading Strategies. Some questionnaire items were modified to adapt to Chinese linguistic features in reading as the writing systems of Chinese and English differ greatly in their orthographic representations, morphology and syntax. Sample items were “I used Chinese radical knowledge to guess meanings of unknown words in the text.” and “I used a known character to guess the meanings of the unknown characters within a word.” The cognitive strategies in the questionnaire, measuring the frequency of actions used by students to solve the tasks in reading, include three subsections: comprehending, memory and retrieval. The metacognitive strategies, measuring the frequency of mental activities that students use to manage and regulate the reading process, contain three subsections: planning, monitoring and evaluating.

All questionnaire items were presented in both English and Chinese. The questionnaire items were first translated into Chinese, then back-translated into English. Two Chinese faculty members verified the accuracy of the translation. After piloting the instrument among 175 L2 learners studied in universities in mainland China, who had similar Chinese language proficiency as the main study participants, the final version of the questionnaires (see Supplementary Appendix B) contained 47 strategies, 6 items for motivational strategies, 20 for cognitive strategies, and 21 for metacognitive strategies. All items used a 6-point Likert scale, ranging from 0 (never) to 5 (always), which indicated an increased frequency of strategy use in L2 Chinese reading.

#### Reading Comprehension Test

Chinese reading comprehension was measured using the reading subtest of HSK Level 5, which was designed to assess intermediate-level learners’ Chinese reading proficiency (Chinese Language Council International and Confucius Institute Headquarters, 2009). With six difficulty levels, HSK is the only recognized large-scale standardized Chinese proficiency test for L2 learners in mainland China (Chinese Language Council International and Confucius Institute Headquarters, 2009). The reading subtest of HSK Level 5 is used to assess reading comprehension ability for understanding literal and inferential information. The reading subtest contains 45 multiple-choice questions, consisting of 15 items for gap-filling, 10 for long passage comprehension, and 20 for reading comprehension (Chinese Language Council International and Confucius Institute Headquarters, 2009).

Based on Pearson and Johnson’s (1978) taxonomy of reading questions, two Chinese reading specialists were invited to classify the 45 test items into literal and inferential questions. Literal questions included identifying the details directly stated in the text. Inferential questions covered inferring the meanings of the words or sentences used in the text, understanding ideas implied in the text, and drawing conclusions based on information stated in several sentences across the text. The ratio of consistent classifications to the total number of classifications reached 94% agreement between the two reading specialists. Disagreements about classifying a question were discussed until consensus was reached. In the end, literal questions contained 7 test items from gap-filling, 10 from long passage comprehension, and 9 from reading comprehension. All other items were categorized as inferential questions.

### Data Collection

The participants first completed a 45-minute reading comprehension test and then filled out a 15-minute strategy use questionnaire. Before the test was administered, students were informed that the data collected would be kept confidential and strictly used for research purposes. The participants joined the study on a voluntary basis and their consent informs were obtained. A dichotomous scoring was used to assess the reading comprehension test items, one point for each correct answer and zero for wrong items. Questionnaire items were scored on the 6-point Likert scale of frequency.

### Data Analyses

Descriptive statistics were first calculated for items in the questionnaire and the reading test. Exploratory factor analysis (EFA) was then conducted using principal axis factoring with oblimin rotation to explore the factor structures of strategies. The oblimin rotation was used because potential factors of motivational and learning strategies were correlated based on the theories and results from previous empirical studies (Oxford, 1990, 2011; Wolters, 1999, 2003; Pekrun, 2006). Composite variables were generated at factor levels for both instruments. This approach, known as item parceling, is commonly used in studies of modeling latent variables in the language education field (Purpura, 1999; Zhang and Zhang, 2013). An aggregate score is more representative of the measured construct, and more statistically reliable than individual items (Little et al., 2002). Confirmatory factor analysis (CFA) was conducted to examine if the identified factor structures and the reading test were good. Structural equation modeling (SEM) was later performed to explore the relationships between motivational strategies, language learning strategies, and literal and inferential comprehension. SEM is an analytic approach for testing hypothesized relationships among observed variables and/or latent factors to determine the degree to which the hypothesized model fits the sample data (Kline, 2011). To evaluate the model fit, several goodness-of-fit indices (*x*^2^/*df* ≤ 3, RMSEA0 ≤ 0.06, SRMR ≤ 0.08, CFI ≥ 0.90, GFI ≥ 0.95) were adopted (Hu and Bentler, 1999; Bentler, 2005). Descriptive and EFA analyses were performed using SPSS 24 (International Business Machines (IBM), 2016) and SEM was conducted via AMOS (Arbuckle, 2010).

## Results

### Descriptive Statistics

Table 1 displays the descriptive statistics of each composite variable, including means, standard deviations, and internal consistency reliability. EFA generated seven factors for 47 questionnaire items measuring L2 learners’ strategy use in the reading test. These seven factors, namely, comprehending (COM), memory (MEM), retrieval (RET), planning (PLA), monitoring (MON), evaluating (EVA), and motivational (MOT) strategies, were labeled regarding strategy taxonomies in L2 learning (Oxford, 1990, 2011; O’Malley and Chamot, 1990; Sheorey and Mokhtari, 2001). Factors of COM, MEM, and RET were hypothesized to measure cognitive strategies, whereas factors of PLA, MON, and EVA were hypothesized to measure metacognitive strategies. Table 2 displays the correlations between factors generated from the questionnaire. Composite scores were generated at factor levels for strategy use questionnaire and reading comprehension test. All the questionnaire item scores for one factor were added up and then divided by the number of items under that factor. Literal and inferential comprehension scores were generated by adding up all the test items’ scores to those two levels of reading comprehension. Table 3 presents the correlations between factors of literal and inferential comprehension. The means of all the questionnaire items were all above the mid-point of the six-point Likert scale. The average scores for literal and inferential questions ranged from 4.46 to 7.64. The reliabilities of all the observed variables ranged from 0.62 to 0.83, indicating acceptable reliabilities. One item in long passage comprehension in the reading comprehension test was dropped because it reduced the reliability of its relevant factor and negatively correlated with other items within the factor.

**TABLE 1:** Means, standard deviations, internal reliability, and sample items for composite variables (*N* = 547).

Composite variable	No. of items	Mean	*SD*	Reliability (Cronbach’s α)	Sample item
COM	7	3.22	0.60	0.77	I tried to understand the content of the text without looking up every word.
MEM	7	3.20	0.64	0.72	I paraphrased or simplified the information in the text to remember.
RET	6	3.22	0.70	0.79	I guessed the meanings of unknown words using root words.
PLA	6	3.05	0.67	0.77	I planned what to do before I began to read texts.
MON	7	3.37	0.65	0.78	I knew when I should read more carefully during the reading.
EVA	8	3.13	0.60	0.83	I checked to see if my understanding of the text was supported by evidence available in the text.
MOT	6	3.61	0.69	0.79	I motivated myself to complete the reading test even if I found it was difficult.
LQGF	7	4.46	1.77	0.62	–
IQGF	8	5.42	1.95	0.62	–
LPC	9	7.64	1.59	0.63	–
LQRC	9	6.85	1.97	0.69	–
IQRC	11	7.58	2.48	0.72	–

*COM, comprehending; MEM, memory; RET, retrieval; PLA, planning; MON, monitoring; EVA, evaluating; MOT, motivational strategies; LQGF, literal questions in gap-filling; LPC, long passage comprehension; LQRC, literal questions in reading comprehension; IQGF, inferential questions in gap-filling; IQRC, inferential questions in reading comprehension.*

**TABLE 2:** Correlation matrix of the strategy use factors.

	COM	MEM	RET	PLA	MON	EVA	MOT
COM	1.00						
MEM	0.57**	1.00					
RET	0.58**	0.57**	1.00				
PLA	0.54**	0.48**	0.49**	1.00			
MON	0.59**	0.50**	0.57**	0.54**	1.00		
EVA	0.63**	0.58**	0.58**	0.66**	0.62**	1.00	
MOT	0.49**	0.44**	0.46**	0.39**	0.63**	0.48**	1.00

COM, comprehending; MEM, memory; RET, retrieval; PLA, planning; MON, monitoring; EVA, evaluating; MOT, motivational strategies. **p < 0.01.

**TABLE 3:** Correlation matrix of the reading comprehension factors.

	LQGF	IQGF	LPC	LQRC	IQRC
LQGF	1.00				
IQGF	0.62**	1.00			
LPC	0.53**	0.50**	1.00		
LQRC	0.54**	0.50**	0.58**	1.00	
IQRC	0.59**	0.60**	0.58**	0.61**	1.00

LQGF, literal questions in gap-filling; IQGF, inferential questions in gap-filling; LPC, long passage comprehension; LQRC, literal questions in reading comprehension; IQRC, inferential questions in reading comprehension. **p < 0.01.

### Contribution of Motivational and Language Learning Strategies to Literal and Inferential Comprehension

To answer the research questions, in terms of the influence of motivational, cognitive, and metacognitive strategies on literal and inferential comprehension, SEM was conducted to examine the relationships among these five latent variables. According to the initial SEM model, metacognitive strategies were hypothesized to directly affect cognitive strategies based on theories and empirical results (Purpura, 1999; Phakiti, 2003, 2008; Zhang and Zhang, 2013). Motivational strategies were hypothesized to directly affect both cognitive and metacognitive strategies as prior research found that motivational strategies encouraged L2 learners to flexibly use cognitive and metacognitive strategies to complete specific tasks in the academic setting (Pekrun et al., 2002; Wolters, 2003, 2011). Motivational, cognitive, metacognitive strategies were hypothesized to have an individual effect on literal and inferential comprehension based on previous research (Phakiti, 2003, 2008; Cheng and Dörnyei, 2007; Zhang and Zhang, 2013).

Before SEM analysis, CFA was first conducted to examine whether observed composite variables of strategies and reading comprehension test loaded on their postulated latent factors. The results of CFA model indicated that all the variables were loaded significantly on their designated latent factors. However, a high correlation was found between cognitive and metacognitive strategies (*r* = 0.93, *p* < 0.001), suggesting multicollinearity between these two variables (Kline, 2011). Previous literature showed that cognitive and metacognitive strategies worked concurrently under a unitary construct in reading contexts (Phakiti, 2003; Zhang et al., 2014). Thus, cognitive and metacognitive strategies were merged into one latent variable in this study, namely, language learning strategies. Supplementary Appendix C presents the results of CFA model.

**FIGURE 1:**
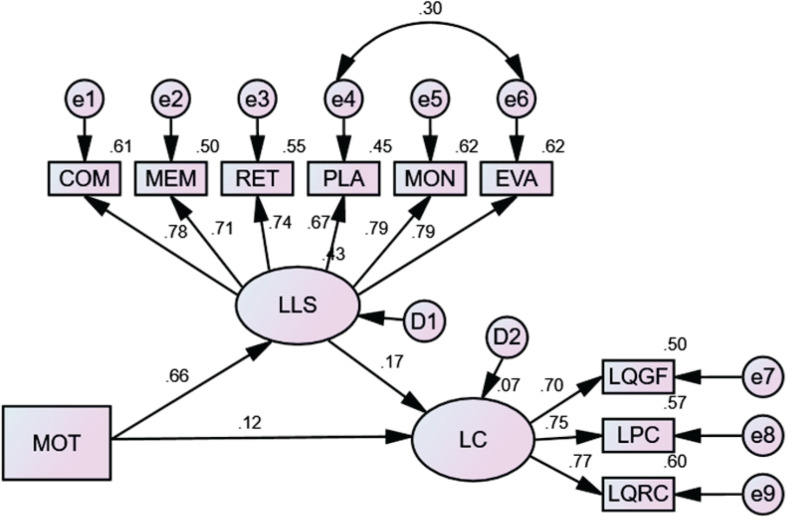
Structural model of the relationships between motivational strategies, language learning strategies and literal comprehension. LLS, Language learning strategies; COM, comprehending; MEM, memory; RET, retrieval; PLA, planning; MON, monitoring; EVA, evaluating; MOT, motivational strategies; LC, literal comprehension; LQGF, literal questions in gap-filling; LPC, long passage comprehension; LQRC, literal questions in reading comprehension.

Structural equation modeling was first performed to explore the relationships between motivational and language learning strategies and literal comprehension. The model was a good fit for the data in this study (*x*^2^/*df* = 2.97, *p* < 0.001; RMSEA = 0.059, SRMR = 0.039, CFI = 0.98, GFI = 0.97). Motivational strategies had a significantly positive effect on language learning strategies (β = 0.66, *p* < 0.001). Language learning strategies had a significantly positive effect on literal comprehension (β = 0.17, *p* < 0.05). Although motivational strategies did not have a significant direct effect on literal comprehension, they had a significant indirect impact on literal comprehension through the mediation of language learning strategies (β = 0.11, *p* < 0.05). Therefore, language learning strategies had fully mediated the positive effect of motivational strategies on literal comprehension. Figure 1 presents the final structural model for motivational strategies, language learning strategies, and literal comprehension.

**FIGURE 2:**
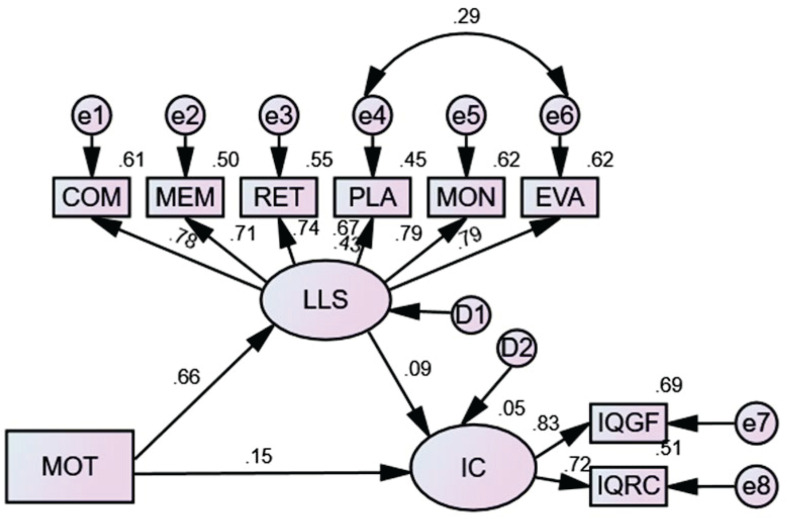
Structural model of the relationships between motivational strategies, language learning strategies and inferential comprehension. LLS, language learning strategies; COM, comprehending; MEM, memory; RET, retrieval; PLA, planning; MON, monitoring; EVA, evaluating; MOT, motivational strategies; IC, inferential comprehension; IQGF, inferential questions in gap-filling; IQRC, inferential questions in reading comprehension.

The second SEM model was conducted to test the relationships between motivational strategies, language learning strategies and inferential comprehension. The model showed an acceptable fit (*x*^2^/*df* = 3.11, *p* < 0.001; RMSEA = 0.060, SRMR = 0.041, CFI = 0.97, GFI = 0.96). As shown in Figure 2, motivational strategies had a significantly and directly positive effect on both language learning strategies (β = 0.66, *p* < 0.001) and inferential comprehension (β = 0.15, *p* < 0.05). However, the direct relationship between language learning strategies and inferential comprehension was not significant (β = 0.09, *p* = 0.20). Motivational strategies had no significantly indirect effect on inferential comprehension (β = 0.14, *p* = 0.23).

## Discussion

This study investigated the relationships between L2 Chinese learners’ motivational and language learning strategies and their reading comprehension at literal and inferential levels. The findings highlight several differences and similarities concerning those reviewed in previous literature.

### Roles of Motivational Strategies, Language Learning Strategies in Literal Comprehension

The result shows that motivational strategies affected L2 learners’ performance on literal comprehension through full mediation of learning strategies, supporting the indirect interactions between motivational strategy use and learners’ learning performance in some previous studies (Wolters, 2003; Schwinger and Stiensmeier-Pelster, 2012). Effective use of motivational strategies enables learners to intentionally activate their willingness and enhance their efforts to facilitate cognitive and metacognitive strategy use, thus optimizing their performance (Zimmerman, 2000; Pekrun and Stephens, 2010). The significant relationships between motivational strategies and learning strategies also highlight the relevance of motivational strategies to cognitive and metacognitive strategies in earlier research (Pintrich and De Groot, 1990). Cognitive and metacognitive strategies, combined as language learning strategies in this study, function as mediator variables between motivational strategies and literal comprehension, indicating motivational strategies alone are insufficient to affect learners’ achievement on literal comprehension. Other strategies are also necessary.

Cognitive and metacognitive strategies were found highly correlated in this study, which is consistent with previous findings that cognitive and metacognitive strategies function concurrently in the reading process (Phakiti, 2003; Zhang et al., 2014). It may be challenging to distinguish cognitive strategies from metacognitive strategies “when they are embedded in complex sequences of behavior or hierarchies of decisions” (Paris et al., 1991, p. 610). When L2 learners try to complete a reading task, they are likely to employ multiple strategies simultaneously to deal with task demands to maximize their comprehension and reading test performance (Zhang et al., 2014; Huang, 2018). The strategies L2 learners use to solve this task usually are not clearly distinguishable, especially when they work under time constraints. Huang (2018) found that successful comprehension in Chinese texts involved a combined use of reading strategies, which contained one or multiple levels of sub-strategies contributing to higher-level strategies. For example, two sub-strategies, using context cues and decoding characters, were applied simultaneously to help readers use a higher-level strategy, inferring words or phrases.

Learning strategies were found to directly affect learners’ performance at literal comprehension. Previous studies found that L2 learners’ strategy use directly impacted their lexical-grammatical ability (Purpura, 1999; Phakiti, 2008). Since lexical-grammatical ability primarily concerns an individual’s competence in literal comprehension, the current finding is consistent with previous research. The direct effect of language learning strategies on literal comprehension indicates that language learning strategies play a crucial role in compensating L2 learners for their lack of knowledge or skills in linguistical processing, such as unknown words and complex sentence structures in the texts. They also support text-based comprehension developed by L2 learners, especially when they encounter a difficult task for which their habitual behaviors in the reading process are insufficient (Cohen, 1998).

### Roles of Motivational Strategies, Language Learning Strategies in Inferential Comprehension

Different from the findings in literal comprehension, motivational strategies directly affected inferential comprehension. No indirect interaction was found between motivational strategies and inferential comprehension, but motivational strategies directly affected language learning strategies. The contextual conditions may lead to different results. The effects of motivational strategies on achievement have been found to be domain-specific (Haag and Götz, 2012; Schwinger and Stiensmeier-Pelster, 2012). The differences are caused by the underlying characteristics of literal and inferential comprehension in this study. L2 learners tend to perceive inferential comprehension tasks as more complicated than literal comprehension. These tasks involve higher-order processing and place more demands on both working memory and cognitive load (Alptekin and Erçetin, 2010). L2 learners may consciously activate and maintain their motivation to engage in a high level of effort in completing a task of inferential comprehension, which later directly affects their reading performance. Otherwise, they would easily give up when encountering difficult questions to measure inferential comprehension in reading, especially with insufficient reading ability.

In terms of literal comprehension, as participants in this study are upper-intermediate learners who are likely to adapt to the demands of Chinese lower-level processes, they may apply motivational strategies with few conscious thoughts. Skilled readers internalize many strategies enhancing reading comprehension as automatic routines when the tasks are not challenging (Alderson, 2000). Therefore, different characteristics of tasks could lead to different effects of motivational strategies. The results emphasize the need to examine the effectiveness of motivational strategies in various contextual conditions.

Language learning strategies failed to affect inferential comprehension. Unlike literal comprehension, inferential comprehension is related to individuals’ higher-level processing skills. L2 learners are required to understand implicit information, connect arguments across the text to identify its main idea, and relate the information in the test to their prior knowledge, thus achieving inferential comprehension (Grabe, 2009; Basaraba et al., 2013). When L2 learners try to complete items to measure inferential comprehension, the role of learning strategies becomes less critical, as it depends on learners’ higher-level comprehension ability. The effects of learning strategies may be minimal, especially when they encounter difficult items beyond their reading proficiency. Although L2 learners’ motivational strategies initiate and sustain their willingness to use learning strategies in this study, without the use of language learning strategies fails to improve their reading performance in inferential comprehension without competence in higher-order processing. Such a result supports the need to explore L2 learners’ strategy use at different levels of reading comprehension, which has been neglected in previous relevant research.

### Roles of Strategy Use in L2 Chinese Reading

Although Chinese reading differs from English reading in cognitive processes and linguistic characteristics (Koda, 2005; Zhou et al., 2018), the effects of strategy use on L2 Chinese reading performance in the work are similar to the results in L2 English studies (Purpura, 1999; Phakiti, 2008). The language learning strategies used by L2 Chinese learners significantly affected their literal comprehension. However, no significant relationship was found between learning strategies and inferential comprehension. Such results indicate that L2 Chinese learners use language learning strategies in lower-level processing to compensate for their lexical and grammatical deficiencies in knowledge. The role of strategy use becomes insignificant in inferential comprehension, especially when L2 Chinese learners’ reading proficiency level fails to reach item difficulty.

It should be noticed that the participants in this study were upper-intermediate learners, which correspond to proficient users of the languages in CEFR. The degree of linguistic disparities between L1 and L2 affects early L2 reading performance, especially for word-decoding. The influence decreases when L2 learners’ reading proficiency improves (Grabe, 2014). These similar findings suggest that L2 learners’ strategy use is not influenced by language-specific elements in L2 Chinese when their L2 reading abilities reach a certain level of proficiency. The findings support Cummins’s (1979) Linguistic Interdependence Hypothesis which suggests that cognitive aspects of language learning, such as reading strategies, can be transferred across languages, even though the writing systems of languages are strongly different.

The results of this study are also consistent with previous L2 English research findings that strategy use only explains a relatively small amount of L2 learners’ reading performance (Phakiti, 2008; Zhang et al., 2014), regardless of the target language. As Bachman (2002) argued, if L2 learners’ language knowledge is below the task’s difficulty level, the influence of strategy use on reading performance will decline.

## Conclusion

This study explored the relationships between motivational strategies, language learning strategies used by L2 learners, and their reading comprehension performance at literal and inferential levels. SEM models indicated that motivational strategies indirectly affected literal comprehension, whereas they directly influenced inferential comprehension. This result highlights the influence of potential contextual differences on the effectiveness of motivational strategies. Language learning strategies were found to significantly affect literal comprehension. However, there was no significant relationship between language learning strategies and inferential comprehension. These results suggest the different roles of learning strategies play in L2 reading depending on the specific levels of comprehension. The relationships between L2 learners’ strategy use and their Chinese test performance found in this study are similar to the results reported in L2 English strategy use research (Purpura, 1999; Phakiti, 2008), indicating that strategy use is not language-specific among proficient L2 learners.

Theoretically, the findings of this study enrich researchers’ knowledge about L2 learners’ strategy use in different levels of reading comprehension, particularly concerning the role of motivational strategies in literal and inferential comprehension. Pedagogically, awareness of the complexity of the motivational and language learning strategies will help Chinese language teachers and L2 learners better understand the effects of strategy use on different levels of comprehension. Teachers are recommended to demonstrate how, when, and why to employ a specific strategy or a group of strategies for a particular reading task to L2 learners, especially motivational strategies that help them to initiate and sustain their willingness to start or complete reading in the challenging tasks (a consideration mostly absent from previous strategy instruction).

This study found that learning strategies were more effective in enhancing L2 Chinese reading performance on items measuring literal comprehension, which was fundamental to inferential comprehension. Given such information, teachers may first give explicit instructions on using strategies for literal questions in Chinese reading, such as strategies facilitating word-decoding and sentence-parsing.

Several limitations of this study need to be noted. First, the questionnaire is the sole instrument used to measure L2 learners’ strategy use. Although a self-reported questionnaire is a viable instrument for collecting and analyzing extensive data with high-reliability levels, participants may over- or under-report on questionnaire items based on their comprehension and the accuracy of their retrieval process in reading (O’Malley and Chamot, 1990). It is recommended to adopt a mixed-methods approach in the future to obtain rich and accurate information on L2 learners’ strategy use in different reading levels. Using qualitative and quantitative methods would allow for cross-validating the roles of L2 Chinese learners’ strategy use in different levels of reading comprehension. Second, the correlation between literal and inferential comprehension is relatively high (*r* = 0.89), indicating that the questions measuring these two levels of comprehension in this study might not be clearly distinguishable. It is suggested to design specific test items based on the characteristics of each level of comprehension in future to further explore the relationships between strategy use and different reading proficiency levels. Third, the participants in this study are all at an upper-intermediate level of Chinese language proficiency. The results can be only applied to that portion of L2 learners with similar reading abilities. Future studies may explore the relationships between strategy use and reading performance at different levels of comprehension among L2 learners with different Chinese reading proficiency levels, e.g., those at the beginning level who are still struggling with word-decoding.

## Data Availability Statement

The raw data supporting the conclusions of this article will be made available by the authors, without undue reservation.

## Ethics Statement

The studies involving human participants were reviewed and approved by the Faculty of Education, University of Hong Kong. The patients/participants provided their written informed consent to participate in this study.

## Author Contributions

LL conceived and designed the study, collect and analyzed the data, and wrote the manuscript. W-IL and ST conceived and designed the study. All authors contributed to the article and approved the submitted version.

## Conflict of Interest

The authors declare that the research was conducted in the absence of any commercial or financial relationships that could be construed as a potential conflict of interest.

## Publisher’s Note

All claims expressed in this article are solely those of the authors and do not necessarily represent those of their affiliated organizations, or those of the publisher, the editors and the reviewers. Any product that may be evaluated in this article, or claim that may be made by its manufacturer, is not guaranteed or endorsed by the publisher.
