# Supplementary material for: Motivational Strategies, Language Learning Strategies, and Literal and Inferential Comprehension in Second Language Chinese Reading: A Structural Equation Modeling Study
Source: Front Psychol. 2021 Aug 12;12:707538. doi: 10.3389/fpsyg.2021.707538 (PMC8397383; doi:10.3389/fpsyg.2021.707538)
Supplement: Supplementary file 1 [file Presentation_1.pdf]

Appendices

Appendix A

Participants by Country

Country	Frequency	Percent
Afghanistan	1	0.2
America	1	0.2
Australia	1	0.2
Azerbaijan	1	0.2
Belgium	1	0.2
Botswana	1	0.2
Brazil	1	0.2
Britain	4	0.7
Bulgaria	2	0.4
Byelorussia	1	0.2
Cambodia	12	2.2
Canada	1	0.2
Chile	2	0.4
Columbia	4	0.7
Congo	1	0.2
Costa Rica	3	0.5
Cuba	1	0.2
Denmark	3	0.5
Ecuador	1	0.2
Emirates	1	0.2
Fiji	1	0.2
France	5	0.9
Germany	1	0.2
Hungary	2	0.4
India	1	0.2
Indonesia	62	11.3
Iran	1	0.2
Israel	1	0.2
Italy	11	2.0
Jamaica	2	0.4
Japan	45	8.2
Jordan	1	0.2
Kazakhstan	16	2.9
Korea	104	19.0
Kyrgyzstan	7	1.3
Laos	24	4.4
Madagascar	3	0.5
Malaysia	1	0.2

Mexico	2	0.4
Mongolia	20	3.7
Nepal	3	0.5
New Zealand	1	0.2
Peru	4	0.7
Philippine	18	3.3
Poland	1	0.2
Romania	2	0.4
Russia	37	6.8
Saudi Arabia	1	0.2
Spain	1	0.2
Sweden	2	0.4
Switzerland	1	0.2
Syria	2	0.4
Thailand	97	17.7
Togo	1	0.2
Turkey	3	0.5
Turkmenistan	2	0.4
Ukraine	4	0.7
Uzbekistan	6	1.1
Vietnam	7	1.3
Yemen	1	0.2
**Total**	**547**	**100.0**

Appendix B

Strategy Use Questionnaire for Chinese Reading

Directions: The purpose of the survey is to collect information about strategies you, as a student of learning Chinese as a second language, used or thought when reading in Chinese. Choose 0 (never), 1(rarely), 2 (sometimes), 3 (often), 4 (usually), or 5 (always) on each statement that best describes how you did or thought during the Chinese reading.

Part A: Cognitive Strategies

Strategy	***Never***					***Always***
1. I tried to identify main ideas in the text.	0	1	2	3	4	5
2. I tried to understand the content of the text without looking up every word	0	1	2	3	4	5
3. I analyzed what the author was trying to say in the text.	0	1	2	3	4	5
4. I made sure I understood hidden meanings in the text.	0	1	2	3	4	5
5. I summarized the main information of the text.	0	1	2	3	4	5
6. I thought about what would happen next while reading.	0	1	2	3	4	5
7. I tried to understand the relationships between ideas in the texts.	0	1	2	3	4	5
8. I drew logical inferences about the text.	0	1	2	3	4	5
9. I translated the texts and the questions from Chinese into my naive language.	0	1	2	3	4	5
10. I paraphrased or simplified the information in the text to remember.	0	1	2	3	4	5
11. I took notes or underline the text while reading to help me remember what I had read.	0	1	2	3	4	5
12. I used typographical features, such as bold face, italics, pictures, tables and figures in the text to identify information and refer to later.	0	1	2	3	4	5
13. I related the information from the text to my prior knowledge about the topic being read.	0	1	2	3	4	5
14. I related the new information with the previous information I had read in the text.	0	1	2	3	4	5
15. I used Chinese radical knowledge to guess meanings of unknown words in the text.	0	1	2	3	4	5
16. I used the knowledge of synonym and antonym to guess the meaning of unknown words in text.	0	1	2	3	4	5

17. I knew which information in the text was more or less important.	0	1	2	3	4	5
18. I guessed meanings of unknown words using root words.	0	1	2	3	4	5
19. I used a known character to guess the meanings of the unknown characters within a word.	0	1	2	3	4	5
20. I used knowledge of prefixes and suffixes to guess the meaning of unknown words.	0	1	2	3	4	5

Part B: Metacognitive Strategies

Strategy	***Never***					***Always***
1. I planned what to do before I began to read texts.	0	1	2	3	4	5
2. I made sure I clarified the goals of the tasks before reading the text.	0	1	2	3	4	5
3. I considered essential steps needed to complete the tasks before I began to read.	0	1	2	3	4	5
4. I took an overall view of the text to see what it was about before reading it.	0	1	2	3	4	5
5. I noticed the characteristics of the text, such as length, organization, sequence of events or procedures.	0	1	2	3	4	5
6. I knew what to do if my intended plans did not work efficiently.	0	1	2	3	4	5
7. I flipped through the reading text before I actually started to do it.	0	1	2	3	4	5
8. I thought about whether the content of the text fitted my reading purposes when I was reading.	0	1	2	3	4	5
9. I checked my comprehension as I moved along the reading.	0	1	2	3	4	5
10. I checked my understanding when I came across new information in the text.	0	1	2	3	4	5
11. I knew when I should read more carefully during reading.	0	1	2	3	4	5
12. I was aware of the time limitation of the reading tasks.	0	1	2	3	4	5
13. I knew how much the texts and tasks remain to be done while reading.	0	1	2	3	4	5
14. I knew when I lost concentration during reading.	0	1	2	3	4	5
15. I noticed when and where I was confused in the text.	0	1	2	3	4	5
16. I immediately correct my misunderstanding in the text when found.	0	1	2	3	4	5
17. I evaluated the accuracy of the information presented in the	0	1	2	3	4	5

	text.						
18.	I checked to see if my understanding of the text was supported by evidence available in the text.	0	1	2	3	4	5
19.	I checked if I understood the texts and tasks.	0	1	2	3	4	5
20.	I evaluated my plans or goals of my reading constantly.	0	1	2	3	4	5
21.	I critically evaluated the information in the text.	0	1	2	3	4	5

Part C: Motivational Strategies

Strategy		*Never*					*Always*
1.	I tried my best to complete the reading task although I may not do it well.	0	1	2	3	4	5
2.	I thought of the desired performance I aimed to achieve.	0	1	2	3	4	5
3.	I motivated myself to complete the reading task even if I found it was difficult.	0	1	2	3	4	5
4.	Even the text was dull and interesting, I managed to work on until I finished.	0	1	2	3	4	5
5.	I made encouraging statements to myself so as to continue to try hard and do my best during reading.	0	1	2	3	4	5
6.	I set goals for the grades I wanted to get in the reading.	0	1	2	3	4	5

Appendix C

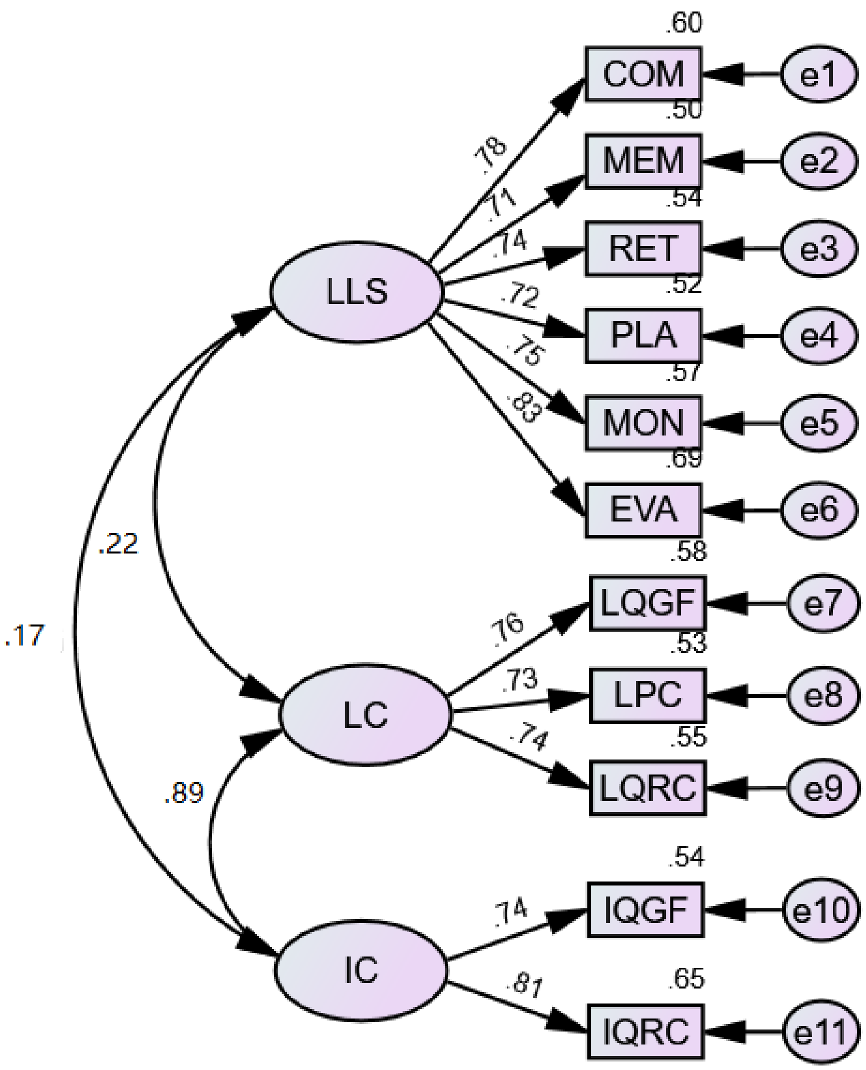

Figure 3. Results for the measurement model

Note. LLS: Language learning strategies; COM: Comprehending; MEM: Memory; RET: Retrieval; PLA: Planning; MON: Monitoring; EVA: Evaluating; LC: Literal comprehension; LQGF: Literal questions in gap-filling; LPC: Long passage comprehension; LQRC: Literal questions in reading comprehension; IC: Inferential comprehension; IQGF: Inferential questions in gap-filling; IQRC: Inferential questions in reading comprehension
